# Circular RNA Expression Profiling and Selection of Key Circular RNAs in the Hypothalamus of Heat-Acclimated Rats

**DOI:** 10.3389/fphys.2019.01112

**Published:** 2019-08-28

**Authors:** Lijun Fan, Gaihong An, Shang Wang, Xuewei Chen, Ying Liu, Zhifeng Liu, Qiang Ma, Jing Wang

**Affiliations:** ^1^Department of Operational Medicine, Tianjin Institute of Environmental and Operational Medicine, Tianjin, China; ^2^Department of Human Movement Science, Tianjin University of Sport, Tianjin, China; ^3^Department of Intensive Care Medicine, General Hospital of Southern Theatre Command of People’s Liberation Army, Guangzhou, China

**Keywords:** high-throughput RNA sequencing technology, circRNAs, heat acclimation, mRNA, microRNA

## Abstract

Circular RNAs (circRNAs) have vital roles in great variety of biological processes. However, expression levels and functions of circRNAs related to heat acclimation (HA) are poorly understood. This study is the first time an in-depth circRNA expression profiling were used to investigate circRNA–miRNA interactions in HA rats in order to further comprehend the mechanisms underlying HA. CircRNA expression profile was performed in rats’ hypothalamus of HA and control group with microarray assays and their functions were predicted by using Bioinformatics analysis. Differential circRNAs and their regulated downstream miRNAs and mRNAs were quantitatively validated by means of quantitative polymerase chain reaction in real-time (RT-qPCR). Enzyme-linked immunosorbent assay (ELISA) was then applied to predict the expression of proteins. In total, 53 circRNAs were expressed distinctively between the HA and Control; up- and down-regulation of circRNAs were 28 and 25, respectively, in HA (fold change > 1.5, *P* < 0.05). Three circRNAs and two miRNAs and three predicted mRNAs were obviously regulated after validated by RT-qPCR in HA rats. Two proteins expression were proportional to their mRNA changes. Further analysis demonstrates that circRNAs closest to HA can be categorized into three signal pathways: including rno_circRNA_014301-vs-rno-miR-3575-vs-*Hif-1*α, rno_circRNA_014301-vs-rno-miR-3575-vs-*Lppr4*, and rno_circRNA_010393-vs-rno-miR-20b-3p-vs-*Mfap4* in hypoxia response pathways, substance/energy metabolism, and inflammatory response pathways. Our findings implicate that many circRNAs regulate expressions of genes that interact with each other to exert their functions during HA.

## Introduction

Heat acclimation results in a multitude of positive adaptations, including elevated whole-body sweat rate and body plasma volume in conjunction with reduced core temperature and heart rate (Schwimmer et al., 2004; Dervisevik et al., 2011). Hypothalamus is a portion of the brain which acts as a temperature-regulating center; the hypothalamic preoptic area (POA) is the primary thermoregulatory region, while other regions of the hypothalamus take part in the maintenance of the afferent and efferent neural pathways, assisting in the governing of core and skin temperature by regulating the surrounding thermo effectors. The hypothalamus is the key temperature sensor, which plays an important role during HA (Matsuzaki et al., 2017). Therefore, we focused on the hypothalamus for understanding the mechanisms underlying HA. To date, physiological effects of HA were well-documented; however, the mechanism of HA at the molecular level HA remains limited. Temperature threshold for thermal injury is generally associated with the increment of inducible cytoprotective networks which includes several components of essential acclimation, such as HIF-1, HSP70, and HSF1. Moreover, neuromodulation of thermoregulation reportedly involves gene expression reprogramming through constitutive downregulation of the genetic involved in food intake and energy metabolism, downregulation of numerous gene transcription due to the maintenance of cellular homeostatic processes, and upregulation of genes associated with immune responses (Schwimmer et al., 2006). In the same vein, Schwimmer et al. (2006) have examined mRNA expression in HA, heat-stressed rats using a cDNA array of stress genes and substantiated (in addition to changes in HSPs) enhancements of antiapoptotic and antioxidative networks by demonstrating the upregulation of gene transcripts associated with cell cycle in the hypothalamus of long-term HA rats (Horowitz et al., 2004). At present, there is limited information regarding molecular mechanisms underlying HA and division of specific essential HA pathways.

Circular RNAs are also known as non-coding RNAs which are abundant during post-transcriptional processes in the brains of eukaryotic organisms (Salmena et al., 2011). Generated through the formation of a covalent bond linking the 3′- and 5′-ends of RNAs via back-splicing, they are not only more stable than linear RNAs, but also exhibit spatiotemporal properties (Chen et al., 2017; Wang et al., 2017). Owing to their capability to sequester miRNAs, circRNAs have pivotal roles in the fine-tuning of post-transcriptional regulation of gene expression (Lukiw, 2013).

Although circRNAs have been studied and reported in many diseases, they have not been studied under conditions of high temperature and heat adaptation. CircRNAs act via the translation of mRNAs into functional proteins under specific conditions. They act as mRNA traps and regulate protein expression through the sequestration of mRNA translation start sides (Li T.R. et al., 2017). CircRNAs and mRNAs may play positive roles in the regulation of HSPs, thereby affecting HA. Furthermore, high temperature can easily trigger a systemic inflammatory response; HA achieved through heat shock response can effectively reduce the production of inflammatory cytokines to reduce inflammatory response (Arieli et al., 2003). CircRNAs take part in the regulation of cell signaling pathways that transfer external stimuli into cells through the cell membrane and trigger a cascade of enzymatic reactions with specific effects, such as gene expression, cell division, and cell apoptosis, among others. Some circRNAs may be involved in inflammatory diseases or immune disorders of the nervous system, and their ability to act as miRNA sponges can be exploited to achieve gene regulation (Li T.R. et al., 2017). CircRNAs may be involved in various immunobiological processes, and their role in regulating cell signaling pathways may affect inflammatory responses to HA. So, circRNAs may affect HA by affecting the regulation of nervous and immune responses.

The hypothalamus is a key brain region that centrally coordinates multiple endocrine and nervous system homeostasis functions, such as body weight and metabolism, osmotic and water balance, and the response to stress, and plays a central role in regulating systemic energy homeostasis and appetite. Exploration of the involvement of circRNA in hypothalamic gene expression and function could better understand its complex role as integrator of the neuronal and endocrine systems and drivers of homeostatic processes (Wang Y. et al., 2016).

In the present study, we hypothesized that circRNAs are associated with HA. In order to test this hypothesis, we determined circRNAs with differences in expression between the HA and control groups using microarray analysis. Using the information obtained from microarray analysis, we aimed to identify the regulatory genes and pathways as well as their targeted genes involved in HA to obtain the in-depth understanding of molecular mechanism underlying HA, which can serve as an experimental basis for the targeted activation of HA.

## Materials and Methods

### Animals and Experimental Conditions

All animal (rat) experiments were performed in accordance with the National Institutes of Health guide for the care and use of Laboratory animals (NIH Publications No. 8023, revised 1978) and approved by the Ethics Committee for Animal Experimentation by Tianjin Institute of Environmental and Operational Medicine. Sprague Dawley (SD) a total of 28 male rats weighing 180–200 g were maintained under a free-feeding schedule and a 12 h–12 h (light from 6:00 to 18:00 h) light–dark cycle. Rats were randomly distributed into HA or C group. HA group was maintained in chamber temperature of 35 ± 1°C with a relative humidity of 55 ± 5% 2 h daily for 30 days to induce homeostasis and their body weight Tre changes were monitored (Dervisevik et al., 2011; Domazetovska et al., 2011; Alexander-Shani et al., 2017). Tre was measured under the arousal condition of food and water *ad libitum*. To avoid gene expression changes due to circadian rhythm, entire experiments were only performed between 14:30 and 16:30. Exposure using an animal heat simulation module (product model: CY-1.7/1500-W; Yantai Hongyuan Oxygen Co., Ltd., China). Body weight were measured every 10 days before and after HA by using a digital balance (Jingsu, Tong Jun) and a thermal probe (1529 CHUB E 4 Thermometer Readout, Fluke Corporation, Hart Scientific Division) inserted 6 cm beyond the rectum, respectively, as previously detailed. Meanwhile, rats in the C group were maintained at a chamber temperature of 24 ± 1°C and fed freely for 30 days. On day 31, rats were sacrificed under pentobarbital sodium anesthesia (100 mg/kg), the entire brain was resected. A vertical incision (2 mm deep) was made at the optic chiasm closely anterior to the mammillary body enabling the removal of the hypothalamus. The isolated hypothalamus which includes the POA then placed in liquid nitrogen. To ensure accurate sampling, each rat was is treated separately, frozen samples for subsequent analysis were stored at 80°C.

### Microarray Hybridization of CircRNAs

The hypothalamus of three rats from each group was used to isolate their total RNA (TRI Reagent^®^ BD, MRC), OD260/OD280 ratio was determined by NanoDrop ND-1000 to determine the total RNA concentration and purity of RNA from each sample. RNA integrity contamination test by denaturing agarose gel electrophoresis. The isolated RNA was stored at −80°C for further experimental verification. Sample preparation and gene chip hybridization were performed by following the Arraystar standard protocol. In short, total RNA of each sample was digested using Rnase R (Epicentre, Inc., United States) which removes all linear RNAs and enrich the circRNAs. Next, the enriched circular RNAs were amplified and transcribed into fluorescent cRNA utilizing a random priming method (Arraystar Super RNA Labeling Kit; Arraystar). The labeled cRNAs were then crossed with Arraystar Rat circRNA Array (8 × 15K, Arraystar). After washing, the arrays scan uses the Agilent Scanner G2505C (Agilent p/n G2565BA, United States).

Agilent Feature Extraction software (version 11.0.1.1) was used to analyze the microarray images. Normalization and subsequent data processing of quantiles using R software’s limma package while Volcano plot filtering was used to identify the circRNAs with significant difference between HA group and C group. Differentially expressed circRNAs between two groups were determined by folding change filtration. Hierarchical clustering was carried out to demonstrate the expression pattern differences between circRNAs of the HA and C groups.

### Bioinformatics Data Analysis

Genes encoded by the circRNAs were identified using circBase^1^ miRNA–mRNA interaction network. The predicted target genes were then input into DAVID^2^ online tool for gene ontology (GO) analysis. KEGG pathway enrichment analysis was performed on the target genes^3^. This analysis returns a *P*-value (EASE-score, Fisher *P*-value, or hypergeometric *P*-value) for each enriched pathway, which denotes the significance of correlation between the pathway and disease condition. Lower *P-*values indicated higher pathway significance, and a *P-*value cut-off of 0.05 was used.

### Data Mining Analysis and Sequence Analysis of miRNA Response Elements

Prediction of circRNA–miRNA interactions was made using Arraystar’s home-made miRNA target prediction software (Pasquinelli, 2012). The annotation in detail of all differentially expressed circRNAs using circRNA–miRNA interaction information. Analysis of miRNA response element (MRE) domains of circRNAs was performed using the Adobe Reader. The association between differential expressions of circRNAs and miRNAs was used to derive miRNA–circRNA interaction networks, and these putative interactions were evaluated on the basis of only perfect seed wobble pairing without any gap as a “strict” parameter. Between expressed miRNA and a target circRNA for a hit was evaluated, the score of more and equal than 140 was considered as a perfect seed match. Evaluation of target miRNAs of circRNAs interactions based on conserved seed-matching sequences was theoretically predicted by means of target Scan and databases. Significantly differentially expressed circRNAs which is defined as having FCs ≥ 1.5 and *P*-values ≤ 0.05 were retained for further analyses. Next, analyses results were filtered, and the circRNAs expressing the difference is sorted according to the folding changes of the circRNAs and the *P-*values. Overall, 15 highly expressed circRNAs were selected for target gene prediction based on their predicted miRNA-binding sites. Literature review and analysis were performed on the predicted target genes with prediction scores of 93–100. Finally, the mRNAs and circRNAs highly correlated with HA, and were selected for RT-qPCR validation.

### RT-qPCR Validation of CircRNAs and miRNAs and Target mRNAs

Each group for three of their respective RNA samples was subjected to circRNAs verification using RT-qPCR (in triplicate). Following TRIzol Reagent (Invitrogen, Carlsbad, CA, United States) manufacturer’s instructions, single-step total RNA extraction was performed on three hypothalamic samples in each experimental group and subsequently the integrity of RNA was detected by denaturing agarose gel electrophoresis. Total RNA was reverse transcribed to synthesize cDNA, and cDNA was amplified for the circRNAs and mRNAs RT-qPCR analysis using the SuperScript^TM^ IV First-Strand Synthesis System and Powerup^TM^ SYBRTM Green Master Mix (Invitrogen Trading Co. Ltd., Shanghai, China), following the manufacturer’s instructions. cDNA was amplified for the miRNAs RT-qPCR analysis using the RNasin Inhibitor, M-MLV reverse transcriptase, 10× RT buffer solution (Epicentre, Inc., United States), dNTP Mix, 2.5 mM each (HyTest Ltd.) and RT primers (Bioligo), as specified the manufacturer’s instructions. RT-qPCR was done using ViiA 7 Dx real-time PCR system (Applied Biosystems), going along with the manufacturer’s instructions. Divergent primers (in preference to commonly used convergent primers) were intended for circRNAs, miRNAs, and mRNAs chosen for further validation by RT-qPCR. Each circRNA and target mRNA was detected in three technical replicates. Using GAPDH as the normalization control for circRNAs and mRNAs RT-qPCR analysis, and U6 as the normalization control for miRNAs RT-qPCR analysis. Detection of PCR products by insertion of the fluorescent dye SYBR Green. The sense and antisense primers used for GAPDH amplification were 5′- GGTGGACCTCATGGCCTACA-3′ and 5′-CTCTCTTGCTCTCAGTATCCTTGCT-3′, respectively. The sense and antisense primers used for U6 amplification were 5′-GCTTCGGCAGCACATATACTAAAAT-3′ and 5′-CGCTTC ACGAATTTGCGTGTCAT-3′, correspondingly. Primers used for the amplification of circRNAs and target gene cDNAs are detailed in Table 1A and miRNAs are detailed in Table 1B. The incident of single peak in the melting curve indicates the specificity of primer.

**TABLE 1:** **(A)** The primer of the validation of differentially expressed circRNAs and target mRNA base sequence and **(B)** The primer of the miRNAs base sequence.

**Regulation**	**Primer name**	**Sense primer (5′–3′)**	**Antisense primer (5′–3′)**	**Base number**
**(A)**				
Up	circRNA_011190	CATCATGGCTGGATCCACGAC	GAGGCTTTGGAGTGCCGTAAT	21
Up	circRNA_014301	GAGTCGCTCCTGCACGAGTTG	GCTGGCATTCTTGCAGTACCG	21
Up	circRNA_016353	GAGGTTGTGAACAGGCTTGAT	GCGTTCCTTTCTTCAAACTCC	21
Up	circRNA_003259	TACACAGCACCGAGATACCAG	GTACTCTTTGGAGCACCAGGC	21
Up	circRNA_002985	AGAACTCGATGCTATCATGGG	CTGCACTGAAGGGCCAAGTAT	21
Down	circRNA_010393	TTCGAGGCCAGTGTAGCGTTG	CCTTGCTCTGTCTGGGGCTAG	21
Down	circRNA_009879	GGTGTCAGTGCTGGTGATGTG	AAAGACACCAGTTTCCGACAG	21
Down	circRNA_005414	AATTGAAGATCGGCTGCATGG	AGTGCCATCGATAAACCACTG	21
Down	circRNA_002960	GCTATGAGGCCTCCCTGGATAC	AAAGGCTCCACGGTGAAAGG	21
Down	circRNA_37695	AATCCGGAAGGAAGCTTGCTG	CCAGACTGCATTCCGTGTCAT	21
	*Fabp3*	TGGGAGTAGAGTTTGACGAGG	CCCGTCCCACTTCTGCACATG	21
	*Cd93*	GATTCCAGAGCGAGCAGAGAG	AGTGGCAGCTGACTATGTTTC	21
	*Dusp7*	AATCCCCATCTCTGACCACTG	TGCTAAGCAGTGCACCAAGAC	21
	*Lppr4*	ACCCAAGAGGCCATTCCATTC	AGAGGCAGCAGTACAGAATCC	21
	*Stard13*	CATTACCAGACACAGGGGACC	GGCTTTCTATAGACAGTGCCT	21
	*Hif-1*α	CACAACGTGAGCTCCCATCTT	AGGGCTTTCAGATAAAAGCAG	21
	*Helz2*	GGTGAAGAGCCGGAGAAACTG	CCCTCCCGACTTATCATGGAG	21
	*Midlip1*	TTCAAGCAAGCAGCCAAGAAC	CTGGCAAAAGTGCATCTGTTC	21
	*Mfap4*	GATTCAACGGCTCAGTGAGTT	TTCTGCTTCAGTGTCAGGAGG	21
	*Hnrnpa3*	ATTTTGAGAAATGGGGCACAC	AGTAGGTCACAAAACCAAAGC	21
	*Lin28b*	GAGCACTGAGAACCAGGGGAT	CTGCTCTAAGCTTTGGCTCAC	21
	*Cand1*	GAGCGCTTCGTACCACATCTC	TCGCTGTCATCATCCAGCTTG	21
	*Mlxip*	GGAAGAGTCGCATCGAGATTG	CCGTGTTTATGCCAATACAGC	21
	*Sec31a*	GCCCAGAACCACCCCATTTAC	TCTCAAGGGAAGCGTTGGTAC	21
	*Zbtb20*	GTGCTGAGAGTCTCCCAGTCG	GAATCCTGGATGCCTGGGAAC	21
	*Hyou1*	GCCTTGGTGGCTGTCCTCTTG	CTTGACAATGGCCACCTTCAT	21
	*Lphn3*	CCACCTCAGCTGTTCATCCTC	TGTGCTCAGCATGTCGCAGTG	21
	
**miRNA name**	**Gene-specific primer (5′–3′)**	**Reverse primer (5′–3′)**	**Length of product (bp)**
**(B)**			
rno-miR-23a-5p	ATTGGGGTTCCTGGGGAT	GTGCGTGTCGTGGAGTCG	89
rno-miR-20b-3p	GGGGTTCTGCAGTGTGAGC	GTGCGTGTCGTGGAGTCG	63
rno-miR-3561-3p	GGGGTTATCCAGGGTAGACA	GTGCGTGTCGTGGAGTCG	65
rno-miR-3575	GGGGAGAACTGCTGGGTAA	GTGCGTGTCGTGGAGTCG	64

In total, a selection of 10 circRNAs, 4 miRNAs, and 17 mRNAs was used for additional validation of the microarray outcomes using RT-qPCR. Primers for amplification of 10 circRNAs and 17 mRNAs and their product length are detailed in Table 1A, 4 miRNAs and their product length are detailed in Table 1B. Data were shown as FC (2^–ΔΔ*Ct*^).

### Enzyme-Linked Immunosorbent Assay Detection of HIF-1α and MFAP4 Proteins

Hypothalamic sections were homogenized on ice bath in PBS (pH 7.4) containing protease inhibitor mix (Solarbio) using an ultrasonic homogenizer and centrifuged at 5000 × *g* for 10 min at 4°C in order to move the organization of debris. To determine protein concentration, protein assays were performed using the Rat MFAP4 (Mlbio, China) and Rat HIF-1α Elisa Kits (Mlbio, China). Equal number of protein was analyzed using the MPM6 System (United States) following the manufacturer’s instructions. A microplate reader (Bio-Rad X-Mark Spectrophotometer, Hercules, CA, United States) was used to measure absorbance at 450 nm. MFAP4 and HIF-1α concentrations were calculated using the ELISA Calc software (Cornple-Software, Iowa City, IA, United States).

### Statistical Analysis

Data and result collected were then expressed in terms of mean and standard deviation. GraphPad Prism Software Version 5.0 (GraphPad Software, La Jolla, CA, United States) was then used to analyze as well as to visualize the data. Significance of quantitative RT-qPCR validated circRNA expression differences between the HA and C groups was evaluated by applying one-way ANOVA and subsequently *post hoc* Student–Newman–Keuls method using the SPSS Statistics Software Version 22.0 (SPSS, Inc., Chicago, IL, United States). Statistically significant are considered when *P*-values < 0.05.

## Results

### Body Weight and Tre

**FIGURE 1:**
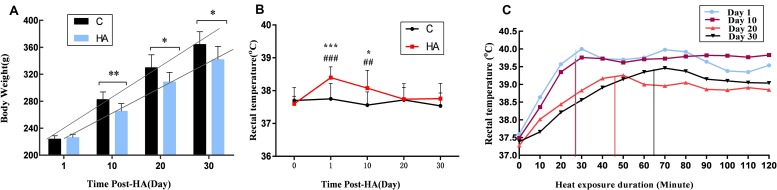
Data are expressed as mean ± SD. HA, heat acclimation (*n* = 17); C, control (*n* = 11). ^∗^*P* < 0.05, ^∗∗^*P* < 0.01, ^∗∗∗^*P* < 0.001 compared with the C group. ^##^*P* < 0.01, ^###^*P* < 0.001 compared with the same group before heat exposure. *P* < 0.05 was considered statistically significant. **(A)** Rats weight in the HA group was significantly lower than C group (*P* = 0.003 for 10 days, *P* = 0.011 for 20 days, and *P* = 0.019 for 30 days). **(B)** Tre in the HA group on days 1 and 10 differed significantly from that in the C group after heat exposure (*P* = 0.000 for 1 days and *P* = 0.025 for 10 days); there was a significant difference of Tre in the HA group after heat exposure on days 1 and 10 compared with the same group before heat exposure (*P* = 0.000 for 1 day and *P* = 0.004 for 10 days). **(C)** During 2-h continuous monitoring, Tre showed an initial rising trend, followed by a decrease and gradual stabilization. There was no difference in Tre between the HA and C groups on day 30.

Changes in body weight and Tre over a 30-day heat exposure period are presented in Figure 1.

The body weight of all rats increased during 30 days HA. After 10, 20, and 30 days of heat exposure, the increase of weight in the HA group was lower than the C group (17.35 ± 0.16) g (*P* < 0.01), (21.31 ± 4.80) g (*P* < 0.05), and (22.65 ± 0.83) g (*P* < 0.05), respectively (Figure 1A). These results are consistent with published studies on HA animals. In the HA group, Tre on days 1 (38.40 ± 0.33°C) and 10 (38.08 ± 0.54°C) was significantly higher than that on day 0 (37.60 ± 0.23°C) after heat exposure (*P* < 0.05). Nevertheless, after day 20 of heat introduction, Tre was restored to its original level before heat exposure. The basal Tre levels of rats in the C group (37.62 ± 0.42°C; Figure 1B) also revealed no significant differences. During the 2-h continuous monitoring, Tre showed a rising trend, followed by a decrease and stabilization. Prolonged heat exposure resulted in a prominent decrease of Tre in the HA group (Figure 1C). These findings are consistent with those of earlier studies conducted on HA rats (Dervisevik et al., 2011; Nakagawa et al., 2016).

### Overview of CircRNA Expression Based on Microarray Analysis

#### Differentially Expressed CircRNAs Based on Microarray Analysis

**FIGURE 2:**
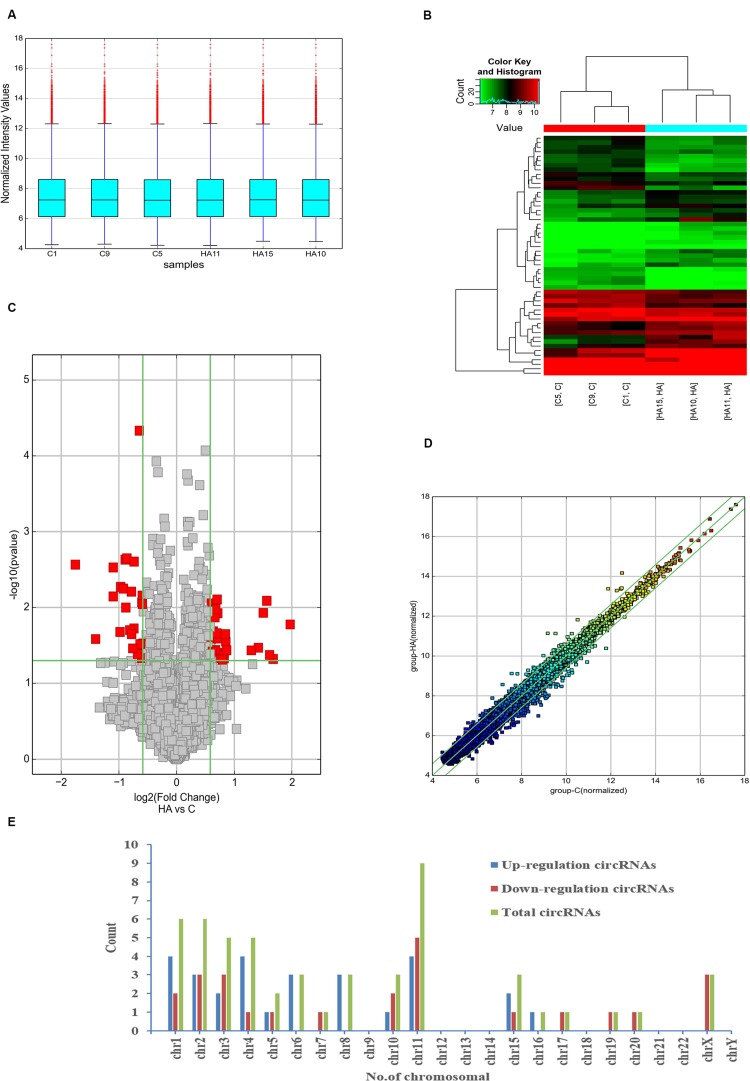
Differential expression of circRNAs in rat hypothalamus. **(A)** Box plot showing the distribution of circRNA expression values in the HA and C groups. **(B)** Hierarchical clustering of circRNAs differentially expressed between the hypothalamus of rats in the HA and C groups; each group contained three individuals (>1.5-fold difference in expression; *P* < 0.05). Expression levels above and below the median expression level were indicated with different colors in all samples. **(C)** Volcano plots constructed using the fold changes (FCs) and *P*-values are shown. Vertical lines correspond to 1.5-FC in the up- and downregulation between the HA and C groups, and horizontal line indicates the *P-*value. The red dot in the figure indicates circRNAs with significant difference in expression. **(D)** Visual assessment of differences in circRNA expression between HA and C using scatter plots. The values corresponding to the *x*- and *y*-axes in the scatter plot are the normalized signals of the samples (Log2 scaled). Green lines indicate FCs. The circRNAs above the top green line and below the bottom green line show a >1.5-FCs between the HA and C groups. **(E)** Chromosomal distributions of differentially expressed circRNAs are shown.

In full, 13,860 circRNA targets in three pairs of samples were detected by the microarray probes. Distributions of circRNA expression values in the HA and C groups after normalization are shown in a boxplot in Figure 2A. Hierarchical clustering of circRNA expression showed noticeable circRNA expression profiles among the HA and C groups (Figure 2B). Volcano and scatter plots were used to visualize the differences between the HA and C groups (Figures 2C,D), and volcano plot screening revealed significant differences in the expression of circRNAs among the two groups. The red points on the left indicate the under expressed circRNAs, and red points on the right indicate overexpressed circRNAs. The two points out of the green lines neighboring the middle of the scatter plots denote FCs cut-off of 1.5 and *P*-values cut-off of 0.05. The chromosomal distributions of differentially expressed circRNAs demonstrated that most circRNAs were transcribed from chromosomes 1, 2, 3, 4, 6, 8, 10, 11, and 15, but seldom from chromosomes 5, 7, 16, 17, 19, and 20 (Figure 2E). Microarray analysis revealed that 53 circRNAs were differentially expressed in the two groups; 28 of these circRNAs were upregulated and 25 were downregulated in the rat model of HA (FC > 1.5; *P* < 0.05). The 10 highest significantly differentially expressed circRNAs are enumerated in Table 2.

**TABLE 2:** Biological information regarding the top 10 upregulated and downregulated circRNAs.

**CircRNA ID**	**FC (abs)**	***P*-value**	**CircRNA type**	**Chromosome**	**Best_transcript**	**GeneSymbol**
**Up-regulation**						
mmu_circRNA_34428	3.9126811	0.0167	Exonic	chr3	NM_017319	*Pdia3*
rno_circRNA_011190	3.1918440	0.0476	Exonic	chr4	NM_053621	*Magi2*
rno_circRNA_014301	3.0545152	0.0420	Exonic	chr6	NM_053888	*Myt1l*
rno_circRNA_016353	2.9556517	0.0081	Exonic	chr8	NM_001108150	*Hmg20a*
rno_circRNA_000931	2.831102	0.0117	Sense overlapping	chr1	XR_145786	*LOC100912383*
rno_circRNA_010071	2.6709626	0.0338	Exonic	chr3	NM_017319	*Pdia3*
rno_circRNA_003259	2.4528114	0.0366	Exonic	chr11	NM_147139	*Itgb5*
rno_circRNA_002985	1.8212257	0.0365	Exonic	chr11	NM_001107109	*Morc3*
rno_circRNA_006302	1.7697922	0.0465	Intronic	chr16	ENSRNOT00000057845	*Dlgap2*
rno_circRNA_011677	1.7347061	0.0428	Exonic	chr4	NM_134376	*Clstn3*
**Down-regulation**						
rno_circRNA_010393	3.3767626	0.0027	Exonic	chr3	NM_001191072	*Ppp1r16b*
rno_circRNA_009879	2.6463196	0.0259	Exonic	chr20	NM_133300	*Ddx39b*
rno_circRNA_005414	2.1433936	0.0071	Exonic	chr15	NM_001033066	*Ddhd1*
rno_circRNA_002960	2.1421682	0.0030	Exonic	chr11	NM_001191662	*Hunk*
rno_circRNA_002958	1.9732566	0.0210	Exonic	chr11	NM_001191662	*Hunk*
rno_circRNA_002957	1.9592926	0.0053	Exonic	chr11	NM_001191662	*Hunk*
rno_circRNA_002959	1.8978736	0.0056	Exonic	chr11	NM_001191662	*Hunk*
mmu_circRNA_37695	1.8574609	0.0023	Exonic	chr5	NM_001015029	*Kpna6*
rno_circRNA_002088	1.6923493	0.0345	Exonic	chr10	NM_130742	*Galnt10*
rno_circRNA_001374	1.5013894	0.0297	Exonic	chr1	XM_217657	*Atrnl1*

#### Bioinformatic Analysis

**FIGURE 3:**
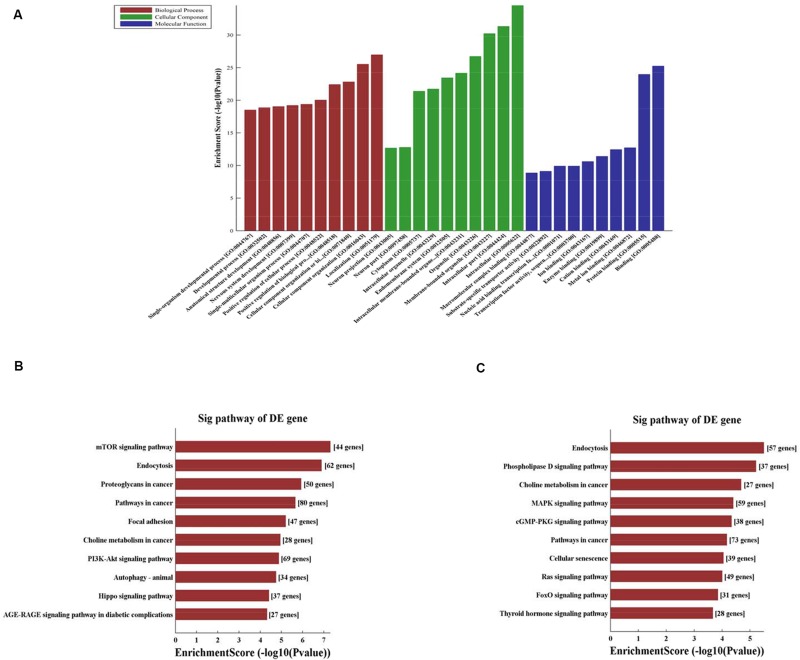
**(A)** Fifteen circRNAs for GO enrichment analysis in terms of BP, CC, and MF. The top significantly enriched target genes and their scores (negative logarithm of *P*-value) are listed as *x*-axis and *y*-axis, respectively. The significant level of GOs are represented in horizontal axis. **(B)** KEGG pathway analysis for 8 up-regulated circRNAs and the top 10 significantly enriched pathways. **(C)** KEGG pathway analysis for 7 down-regulated circRNAs and the top 10 significantly enriched pathways. The significant level of pathways represented in horizontal axis.

CircRNA function is related to the recognized functions of host linear transcripts that in the hypothesis, DAVID to further analyze differentially regulated linear transcripts. The GO project provides major bioinformatics initiatives to define the constant evolving of our knowledge toward gene and how gene encodes to form products attributing to the BP, CC, and MF in any organism^4^. Prediction terms with *P-*values < 0.05 were designated and ranked according to the lowest *P-*values. The top 10 generally GO terms were listed in all comparison groups categorized by BP, CC, and MF and ranked by their *P*-value. Our results indicated that the most augmented and expressive BP terms were localization; CC organization or biological regulation; and positive regulation of biological, cellular, and metabolic processes. Regarding CC, terms were mostly enriched for cell parts, intracellular, organelle, cytoplasm, endomembrane system, and neurons. Moreover, the most enriched and expressive MF terms were in close proximity toward binding, catalytic activity, substrate-specific transporter activity, and transferase activity involved in metabolism (Figure 3A). These findings indicated that in HA rat, highly expressed and enriched host linear transcripts were predominantly functionally engaged in several main processes such as stress response, energy breakdown, and positive ruling of cellular processes. All of above responses indicated an important regulatory function of circRNAs during HA, suggesting that many circRNAs regulated the expression of genes that interact with each other to exert their functions. Furthermore, KEGG pathway enrichment analysis was performed and its significantly enriched pathways (*P* < 0.05) were then selected and ranked according to their *P*-values. The seven highest regulated pathways returned by KEGG pathway enrichment analysis were the phospholipase D signaling pathway, MAPK signaling pathway, mTOR signaling pathway, cGMP-PKG signaling pathway, Ras signaling pathway, PI3K-Akt signaling pathway, AGE-RAGE signaling pathway, and autophagy and endocytosis (Figures 3B,C).

### Construction of the CircRNA–miRNA–mRNA Coexpression Network

**FIGURE 4:**
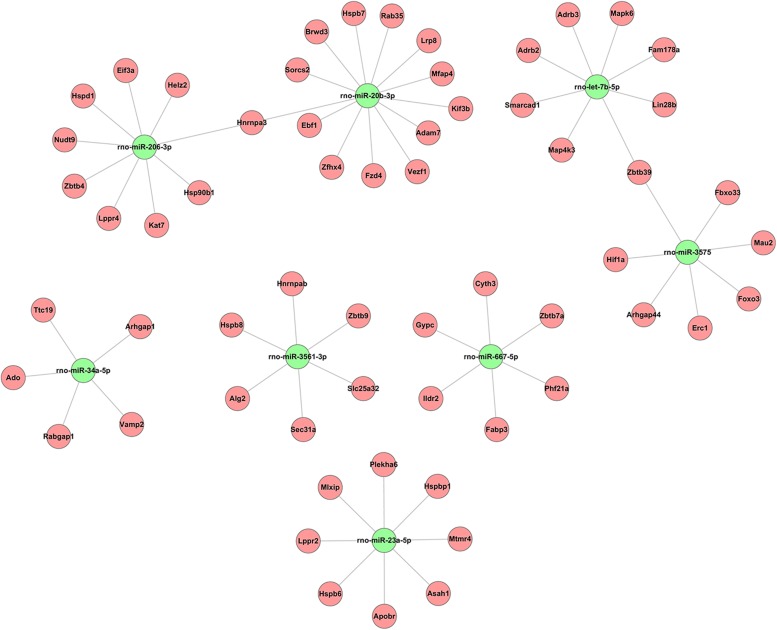
Based on sequence-pairing prediction predicted 15 circRNAs targeted circRNA–miRNA–mRNA/gene network. The results of miRNA-binding sites predicted by mirSVR, and targeted TargetScan and miRanda predicted miRNAs and mRNAs, were considered. The red circles represent mRNAs and green nodes represent miRNAs.

**FIGURE 5:**
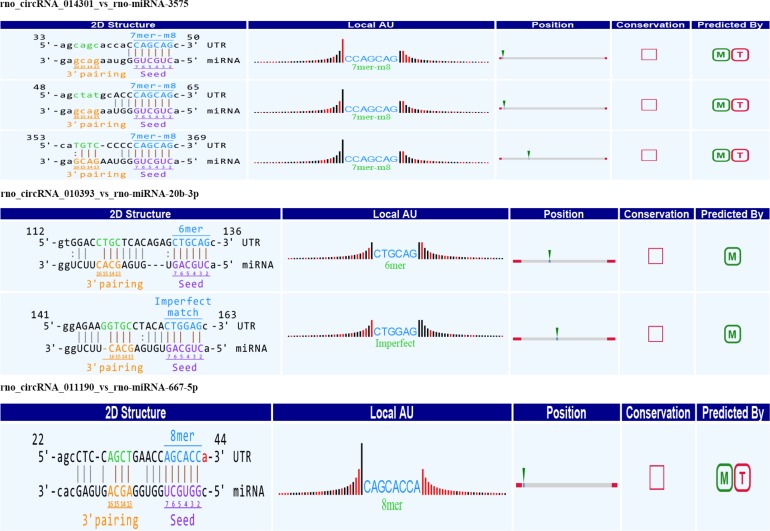
Seed sequence matching predicted the direct interaction of rno_circRNA_014301, rno_circRNA_010393, and rno_circRNA_011190 with the three miRNAs: rno-miR-3575, rno-miR-20b-3p, and rno-miR-667-5p.

Alignment of circRNAs to miRNAs was performed, the public database circBase (http://www.circbase.org) was used to screen for targeted miRNAs and 5 miRNAs with the highest mirSVR scores were identified for each differentially expressed 15 circRNAs using miRNA target-prediction software. The top 5 miRNAs predicted to be the targets of 8 upregulated and 7 downregulated circRNAs are shown in Table 3. Next, we predicted the target genes of the top 75 miRNAs using targetscan7.1 and mirdbV5. We generally accepted the results that overlapped between the two databases and found that the 15 circRNAs targeted 9021 mRNAs. A part of the miRNA-target network with a target gene prediction score ≤ 93 was constructed (Figure 4). Some of the predicted target miRNAs, including rno-miR-20b-3p, rno-miR-23a-5p, and rno-miR-34a-5p, which were downregulated, indicated a role in the regulation of lipid metabolism. Combined with the target mRNA prediction results, we selected genes with target scores of 93∼100 for 200 mRNAs and performed a literature review. The public database circBase (http://www.circbase.org) was used to screen for targeted miRNAs. Seed sequence matching and specific base pairing resulted in the identification of the following miRNAs: rno-miR-3575, rno-miR-20b-3p, rno-miR-23a-5p, and rno-miR-3561-3p. Using circRNA database sources, detailed annotation information for targeted miRNAs can be predicted (Figure 5). CircRNAs can regulate the expression levels of mRNA through a circRNA-miRNA-mRNA pathway. Finally, 10 circRNAs, 4 miRNAs, and 17 mRNAs related to lipid metabolism and immune response, which were highly correlated with HA, were selected for RT-qPCR validation (Table 1). Expression of these genes may facilitate HA.

**TABLE 3:** Predicted miRNA response elements regarding the eight upregulated and seven downregulated circRNAs.

**CircRNA ID**	**Predicted miRNA response elements (MREs)**				
	
	**MRE1**	**MRE2**	**MRE3**	**MRE4**	**MRE5**
**Up-regulation**					
*mmu_circRNA_34428*	*rno-miR-338-3p*	*rno-miR-3551-5p*	*rno-miR-3544*	*rno-miR-107-5p*	*rno-miR-3064-3p*
*rno_circRNA_011190*	*rno-miR-320-5p*	*rno-miR-207*	*rno-miR-483-3p*	*no-miR-667-5p*	*rno-miR-125b-5p*
*rno_circRNA_014301*	*rno-miR-672-5p*	*rno-miR-3593-5p*	*rno-miR-3575*	*rno-miR-1306-5p*	*rno-miR-466b-5p*
*rno_circRNA_016353*	*rno-miR-667-5p*	*rno-miR-206-5p*	*rno-miR-6327*	*rno-miR-293-3p*	*rno-miR-320-3p*
*rno_circRNA_003259*	*rno-miR-667-5p*	*rno-miR-494-5p*	*rno-let-7c-5p*	*rno-let-7b-5p*	*rno-miR-802-3p*
*rno_circRNA_002985*	*rno-miR-1b*	*rno-miR-153-5p*	*rno-miR-1-3p*	*rno-miR-206-3p*	*rno-miR-433-5p*
*rno_circRNA_006302*	*rno-miR-93-3p*	*rno-miR-3084b-3p*	*rno-miR-308-4d*	*rno-miR-3084a-3p*	*rno-miR-203b-3p*
*rno_circRNA_011677*	*rno-miR-6332*	*rno-miR-409b*	*rno-miR-320-5p*	*rno-miR-1297*	*rno-miR-3568*
**Down-regulation**					
*rno_circRNA_010393*	*rno-miR-20b-3p*	*rno-miR-1956-3p*	*rno-miR-762*	*rno-miR-93-3p*	*rno-miR-3593-5p*
*rno_circRNA_009879*	*rno-miR-742-3p*	*rno-miR-6318*	*rno-miR-6326*	*rno-miR-20b-3p*	*rno-miR-221-5p*
*rno_circRNA_005414*	*rno-miR-336-3p*	*rno-miR-760-5p*	*rno-miR-107-5p*	*rno-miR-135a-5p*	*rno-miR-181a-1-3p*
*rno_circRNA_002960*	*rno-miR-488-3p*	*rno-miR-23a-5p*	*rno-miR-653-3p*	*rno-miR-141-5p*	*rno-miR-3561-3p*
*mmu_circRNA_37695*	*rno-miR-3084a-3p*	*rno-miR-3084b-3p*	*rno-miR-3084d*	*rno-miR-3584-5p*	*rno-miR-214-3p*
*rno_circRNA_002088*	*rno-miR-542-5p*	*rno-miR-26b-3p*	*rno-miR-138-5p*	*rno-miR-3084c-3p*	*rno-miR-337-3p*
*rno_circRNA_001374*	*rno-miR-466c-5p*	*rno-miR-582-3p*	*rno-miR-34a-5p*	*rno-miR-628*	*rno-miR-34c-5p*

### Validation of Candidate CircRNAs and miRNAs and mRNAs Using RT-qPCR

**FIGURE 6:**
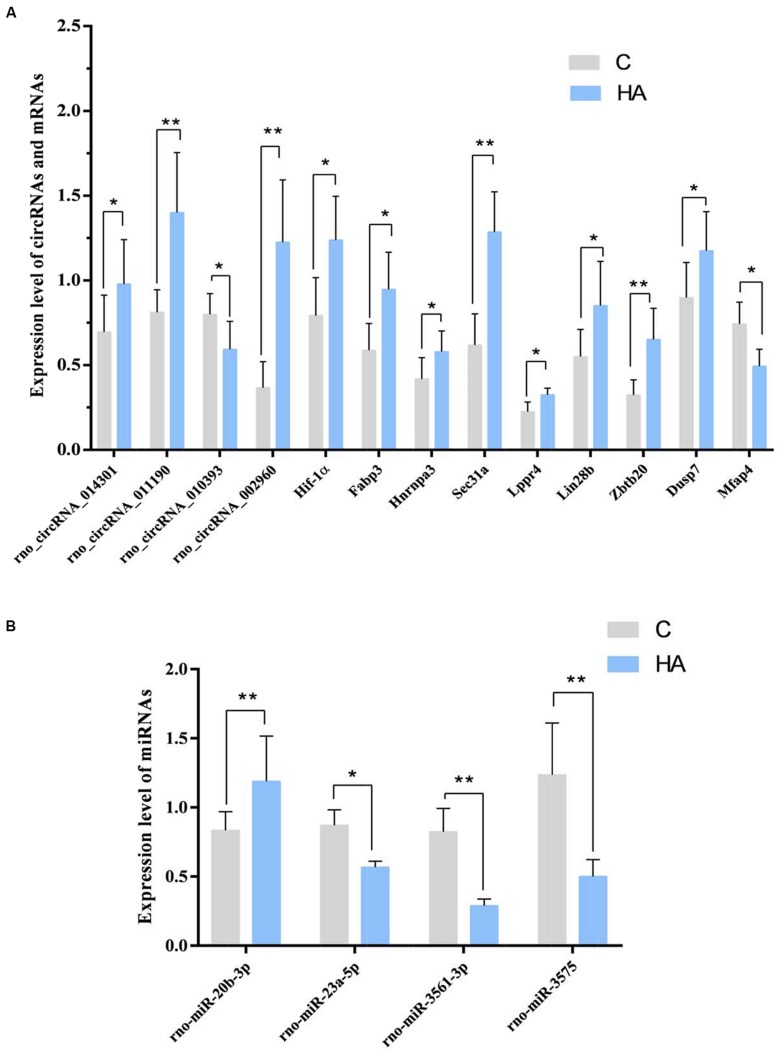
**(A)** GAPDH was measured as a reference gene, and three independent times for each sample of RNA were detected for these circRNAs and mRNAs. ^∗^*P* < 0.05, ^∗∗^*P* < 0.01. **(B)** U6 was measured as a reference gene, and three independent times for each sample of RNA were detected for microRNAs. ^∗^*P* < 0.05, ^∗∗^*P* < 0.01.

Among the 10 circRNAs, only 3 upregulated circRNA and 1 downregulated circRNAs were significant. The expression of rno_circRNA_002960, rno_circRNA_014301, and rno_circRNA_011190 was upregulated 3.34-, 1.41-, and 1.73-fold, respectively (*P* < 0.05) in the HA group compared with that in the C group, whereas expression of rno_circRNA_010393 was downregulated 0.74-fold (*P* < 0.05). The FCs for rno_circRNA_010393, rno_circRNA_014301, and rno_circRNA_011190 were consistent with the results of microarray analysis. Among the four miRNAs, rno-miR-23a-5p, rno-miR-3575, and rno-miR-3561-3p were down-regulated (0.65-, 0.39-, and 0.35-fold, respectively; *P* < 0.05), while rno-miR-20b-3p was up-regulated 1.42-fold (*P* < 0.05). Among the 17 predicted mRNAs, 8 upregulated mRNAs and 1 downregulated mRNAs could be validated. The expressions of *Dusp7*, *Hnrnpa3*, *Lppr4*, *Hif-1*α, *Sec31a*, *Zbtb20*, *Fabp3*, and *Lin28b* were significantly upregulated (1.31-, 1.38-, 1.44-, 1.56-, 2.08-, 2.01-, 1.61-, 1.54-, and 3.34-fold, respectively; *P* < 0.05). Expressions of *Mfap4* was downregulated (0.67-fold; *P* < 0.05; Figure 6). The above target genes were predicted for their close relation to BPs of substance metabolism, energy metabolism, and immune regulation, indicating an important role of these genes during HA. In a word, we confirmed the differential expression of three circRNAs.

In the light of our findings, we speculated that the following four pathways promote HA: rno_circRNA_014301-vs-rno-miR-3575-vs-*Hif-1*α in the hypoxia response pathway; rno_circRNA_ 014301-vs-rno-miR-3575-vs-*Lppr4* and rno_circRNA_010393-vs-rno-miR-20b-3p-vs-*Mfap4* in the lipid/lipoprotein metabolic pathways; and rno_circRNA_011190 may affect the expression of *Fabp3* by regulating rno-mir-667-5p to regulate the lipid metabolism pathway (Table 4).

**TABLE 4:** The three circRNA–miRNA–mRNA pathway to promote HA.

**Pathway**	**CircRNA**	**Expression changes**	**miRNA**	**Expression changes**	**mRNA**	**Expression changes**
1	rno_circRNA_014301	Up 1.41-fold	rno-miR-3575	Down 0.39-fold	*Hif-1*α	Up 1.56-fold
2	rno_circRNA_014301	Up 1.41-fold	rno-miR-3575	Down 0.39-fold	*Lppr4*	Up 1.44-fold
3	rno_circRNA_010393	Down 0.74-fold	rno-miR-20b-3p	Up 1.42-fold	*Mfap4*	Down 0.67-fold

### Validation of HIF-1α and MFAP4 Expressions in the Hypothalamus Using ELISA

**FIGURE 7:**
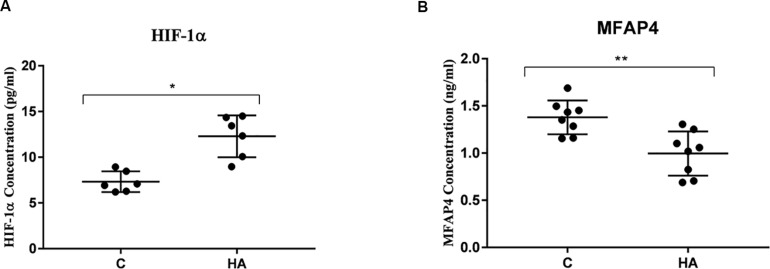
Validation of HIF-1α and MFAP4 as HA-related proteins by ELISA analysis. Data are expressed as mean ± SD. ^∗^*P* < 0.05, ^∗∗^*P* < 0.01. **(A)** The expression level of HIF-1α of HA group was higher than that of control group (*P* = 0.14, *P* < 0.05). **(B)** A significant lower expression level of MFAP4 was found the hypothalamus from HA group (*P* = 0.002, *P* < 0.05).

To ascertain that cell protection and thermally induced cross tolerance definitely occur in HA rat’s hypothalamus, the HIF-1α and MFAP4 protein expression levels were measured (Figure 7). Hypothalamic protein expression level of HIF-1α in HA group is significantly greater than group C (*P* < 0.05), whereas hypothalamic protein expression level of MFAP4 was significantly lower in the HA group than that in the C group (*P* < 0.01). These findings are consistent with the mRNA expression levels of HIF-1α and MFAP4.

## Discussion

Circular RNA is a ubiquitous non-coding RNA which are more stable and with highly conserved sequences. This recently characterized branch of endogenous non-coding RNAs is involved in various cellular as well as developmental processes. These properties make circRNAs appealing as biomarkers. To date, circRNAs and their studies of their functions have been previously reported in cancers such as in the liver, oral, and skin (Memczak et al., 2013; Yang et al., 2016). Thus far, molecular mechanisms underlying HA remain unclear, particularly at the genomic level, and many studies on HA have focused on its basic physiology. However, knowledge regarding the role of circRNAs during HA is limited. In the present study, high-throughput circRNAs microarray assays have been used to identify differentially expressed circRNAs between the hypothalamic tissues of rats in the HA and C groups and identified 13,860 circRNAs. Investigation of the mechanistic roles of circRNAs during HA can provide a platform for rapid evaluation and a scientific basis for further functional validation and elucidation of the genetic mechanisms underlying HA.

### Stable Rat HA State

In the present study, a gradual increase in body weight was seen in the HA group; however, at the end of heat exposure, body weight of HA group was lower than that in the C group. Tre of rats in the HA group significantly increased at the early stages of heat exposure, and it was restored to the original levels after 20 days of heat exposure. However, Tre did not significantly differ among the rats in the C group. Previous studies have demonstrated that after HA achieved through long-term heat exposure, rats in the HA group weighed less than rats in the control groups (*P* < 0.05). During early stages of heat exposure, Tre of rats in the HA group was seen to have a significantly greater value than before heat exposure (*P* < 0.05), and no significant differences in Tre were observed between the groups at the end of heat exposure (Alexander-Shani et al., 2017; Matsuzaki et al., 2017). Our results are consistent with these previous findings and support the fundamental role of autonomic thermoregulation in long-term HA rats. In our rat model, a stable HA state was achieved.

### Bioinformatic Pathways and miRNA Target Prediction Based on Microarray Analysis

In the present study, 53 circRNAs were aberrantly expressed during HA, suggesting their role during HA. Moreover, analysis of chromosomal distributions of differentially expressed circRNAs revealed that the majority of circRNAs were transcribed from chromosomes 1, 2, 3, 4, and 11. HA organisms may exhibit higher lipid peroxidation levels (Coggins et al., 2017). Earlier studies have exhibited that long-term heat exposure of rats resulted in enhanced endurance and remodeling of membrane lipid composition in response to ambient temperature changes. Such remodeling could interfere with the interactions of specific lipids with proteins. Upregulation of muscarinic receptors and regulate the fatty acid environment of these receptors and other proteins yield a key means in protecting their integrity during thermal stress, and these pathways are further involved in other regulatory mechanisms (Shmeeda et al., 2002). These lipid mediators activate their intracellular signaling cascades that promote cell survival through their specific receptors and coupling mechanisms (Shohami and Horowitz, 2013). Therefore, regulation of lipid/lipoprotein metabolism is an important pathway for the regulation of HA. In the present study, in combination with the top five predicted miRNAs for each differentially expressed circRNA, a literature review was conducted; the predicted target miRNAs, which includes rno-miR-20b-3p, rno-miR-23a-5p, and rno-miR-34a-5p, were found to have been downregulated, suggesting their roles in the synthesis of lipids and regulation of lipid metabolism (Zhang et al., 2014; Liu et al., 2015; Shi and Huang, 2015). In GO analysis, we found that three pathways associated with inflammatory responses, such as MAPK, mTOR, and AGE-RAGE (Maya-Monteiro and Bozza, 2008; Cota, 2014; Li et al., 2018; Logan and Storey, 2018). It was reported that the inflammatory response may be closely related to HA formation. In addition, we have also found phospholipase D, cGMP-PKG, PI3K-Akt, and AGE-RAGE signaling pathways (Bruntz et al., 2014; Li T. et al., 2017; Sandoval et al., 2017; Tripathi and Garg, 2018), which may be involved in cell differentiation, apoptosis, and energy metabolism regulation to promote HA formation. Functional assessment of target genes demonstrated that several mRNAs might be part of the circRNA–miRNA–mRNA network, as indicated by the upregulation of HIF-1α and HSPs. According to research reported the temperature threshold for thermal damage is related to the gradual enhancement of the induced cell protection network, and with the remodel of cytoprotective networks changes, such as HSP70, HSF-1, and HIF-1. Expressions of them were upregulated during HA (Maloyan et al., 1999; Arieli et al., 2003; Horowitz and Assadi, 2010). These outcomes are consistent with preceding genomic and proteomic findings concerning HA.

### Three CircRNA–miRNA–mRNA Expression Networks Promote HA

Circular RNAs serve material functions because they regulate gene expression at multiple levels. They directly bind MREs to the shared miRNAs through direct competition, thereby negatively regulating the effect of miRNA on the target mRNA (Hansen et al., 2013; Yang et al., 2016). Accordingly, we hypothesized that the circRNA–miRNA–mRNA axis is the mechanism underlying HA. Based on the hypothesis that circRNA acts as an miRNA sponge that predicts miRNA binding partners, we speculated the involvement of four pathways in HA, including rno_circRNA_014301-vs-rno-miR-3575-vs-*Hif-1*α, rno_circRNA_014301-vs-rno-miR-3575-vs-*Lppr4*, rno_circRNA_010393-vs-rno-miR-20b-3p-vs-*Mfap4*, and rno_ circRNA_011190 may affect the expression of *Fabp3* by regulating rno-mir-667-5p. Interestingly, we observed the upregulation of HIF-1α and downregulation of MFAP4 in the hypothalamus of HA rats. The hypoxia response pathway is intrinsic to HA, and the HIF-1α pathway induces cross tolerance against hypoxia following long-term HA. HA leads to HIF-1α upregulation and faster activation (Tetievsky et al., 2008), and it plays an essential role in an intricate antiapoptotic network during HA (Gultice et al., 2009). Furthermore, several studies have reported elevated serum MFAP4 levels in allergic asthma, liver disease, and liver fibrosis (Wulf-Johansson et al., 2013; Pilecki et al., 2015) as well as the role of MFAP4 in inflammatory mechanisms in the lung (Schlosser et al., 2006). Involved in intercellular interactions and cell adhesion that MFAP4 is an extracellular matrix protein; it can be activated and proliferated by integration-mediated vascular smooth muscle cells and lead to arterial stenosis and proinflammatory effects (Hemstra et al., 2018). In the cerebrospinal fluid of HA rabbits, the matrix protein MFAP4 was highly upregulated (Wang J. et al., 2016), indicating the role of MFAP4 in cell protection and inflammatory mechanisms. In the present study, downregulation of MFAP4 expression could have resulted in the reduction of antigen-induced inflammatory cytokine production by other cell types, thereby enhancing the adaptive response of the body to heat. FABP3 that is expressed in central nervous system has different roles in cell growth, fatty acid transport, gene transcription, and cell signaling (Tang et al., 2016). It may play an essential role in fatty acid transport, cell signaling during HA. LPPR4 is responsible for regulating of neuronal plasticity, and also has important part in the regulation of excitatory neurotransmission (Yu et al., 2015). LPPR4 may promote the formation of HA in regulating nerve excitability. Taken together, we provided an in-depth profiling of circRNA expression in HA rats and posited three representative circRNAs as candidate HA biomarkers. We demonstrated the potential roles of rno_circRNA_014301, rno_circRNA_010393, and rno_circRNA_011190 in energy metabolism and immune regulation associated with HA. Our results are consistent with Horowitz’s points of view, that the HIF-1α antioxidant pathway and HSP anti-apoptotic pathway are enhanced in HA (Horowitz and Assadi, 2010). In addition, the constitutive downregulation of genes involved in food intake and energy metabolism, and a vast amount of genes associated with immune response the obvious upregulation support these conclusions. Downregulation of several gene transcripts associated with homeostatic cellular processes maintenance for cross tolerance in HA was reported (Horowitz et al., 2004; Horowitz, 2007).

## Conclusion

In conclusion, using high-throughput circRNA microarrays, we, for the first time, measured the hypothalamic circRNA expressions in HA rats. We suggested that circRNA levels change to coordinate thermoregulation. Moreover, we identified rno_circRNA_014301, rno_circRNA_010393, and rno_circRNA_011190 as candidate biomarkers for HA, which may regulate HA in rats by controlling rno-miR-3575, rno-miR-20b-3p, and rno-miR-667-5p activity to regulate expression levels of *Hif-1*α, *Lppr4*, *Mfap4*, and *Fabp3*, respectively. These genes are involved in networks critical for hypoxia response pathways, substance/energy metabolism, and inflammatory response pathways to promote HA.

## Data Availability

The datasets generated for this study can be found in the Gene Expression Omnibus https://www.ncbi.nlm.nih.gov/geo/query/acc.cgi?acc=GSE131044.

## Ethics Statement

All animal experiments were carried out in accordance with the National Institutes of Health guide for the care and use of Laboratory animals (NIH Publications No. 8023, revised 1978). And also was approved by the Ethics Committee for Animal Experimentation of the Tianjin Institute of Environmental and Operational Medicine.

## Author Contributions

LF, ZL, QM, and JW: conceptualization. LF, QM, and JW: methodology. LF and GA: validation. LF: formal analysis and writing – original draft. LF, GA, and XC: investigation. SW, JW, ZL, and QM: supervision. XC, YL, and JW: resources. ZL and JW: project administration. JW: writing – review and editing.

## Conflict of Interest Statement

The authors declare that the research was conducted in the absence of any commercial or financial relationships that could be construed as a potential conflict of interest.

## Footnotes

1 http://www.circbase.org

2 https://david.ncifcrf.gov

3 https://www.genome.jp/kegg/

4 http://www.geneontology.org

## Abbreviations

BP: biological process
CC: cellular component
CircRNA: circular RNAs
Dusp7: dual specificity phosphatase 7
Fabp3: fatty acid binding protein 3
FC: fold change
GSP: gene-specific primer
HA: heat acclimation
HIF-1: hypoxia-inducible factor-1
HSF1: heat shock factor 1
HSP70: heat shock protein 70
Lppr4: lipid phosphate phosphatase-related protein type 4
MF: molecular function
MFAP4: microfibrillar-associated protein 4
R: reverse primer
Tre: rectal temperature
Zbtb20: zinc finger and BTB domain containing 20.
